# Outcome of Nivolumab-Induced Vogt–Koyanagi–Harada Disease-Like Uveitis in a Patient Managed without Intravenous Methylprednisolone Therapy

**DOI:** 10.1155/2023/9565205

**Published:** 2023-02-08

**Authors:** Ryoji Nagai, Akinari Yamamoto, Akiko Yoshida, Akiko Mikawa

**Affiliations:** ^1^Department of Ophthalmology, Nishi-Kobe Medical Center, Kobe, Japan; ^2^Department of Ophthalmology and Visual Sciences, Kyoto University Graduate School of Medicine, Kyoto, Japan

## Abstract

**Background:**

In recent years, immune checkpoint inhibitors (ICI) have been often used for several types of cancers. Immune-related adverse events (irAEs) are autoimmune responses caused by ICI. Among the different types of irAEs, uveitis is common in ophthalmology. Moreover, there are reports on Vogt–Koyanagi–Harada (VKH) disease-like uveitis. In most cases, VKH, as in the usual VKH, is managed with intravenous methylprednisolone therapy. *Case Report*. A 72-year-old man was diagnosed with gastric cancer, and he was treated with nivolumab, a type of ICI. After eight cycles of nivolumab therapy, he developed fulminant type 1 diabetes mellitus and diabetic ketoacidosis. Thus, the treatment was discontinued. Subsequently, the patient was referred to our department due to bilateral blurry vision. He had decreased visual acuity in both eyes, and slit lamp examination revealed the presence of bilateral anterior chamber cells and keratic precipitates. Fundus examination showed bilateral serous retinal detachment (SRD), wavy retinal pigment epithelium (RPE), and choroidal thickening. Cerebrospinal fluid examination revealed prominent pleocytosis. Thus, we initiated eye drop therapy and subtenon injection of triamcinolone acetonide on the right eye only. After 1 month, SRD and wavy RPE disappeared, and the patient's visual acuity improved. Further, both eyes had similar improvements in visual acuity and abnormal findings. Oral prednisolone was subsequently administered for hearing loss. However, intravenous methylprednisolone was not used, and ophthalmologic findings and visual acuity did not change before and after systemic steroid therapy. One year after disease onset, SRD and wavy RPE did not relapse.

**Conclusion:**

Nivolumab-induced VKH disease-like uveitis can have good outcomes even in a patient managed without intravenous methylprednisolone therapy.

## 1. Introduction

In recent years, immune checkpoint inhibitors (ICI) targeting programmed cell death 1 (PD-1) have been often used in cancer treatment. PD-1 is a coinhibitory molecule that exists on the surface of T cells and binds to programmed death-ligand 1 expressed on the surface of tumor cells. The binding of ligand and PD-1 inhibits T cell activation leading to apoptosis. Nivolumab, a human immunoglobulin G4 monoclonal antibody, binds to PD-1, maintains T cell activation, and inhibits T cell apoptosis [1]. Since it was approved in 2014, its application has expanded over the years. That is, it is used to manage different types of cancer. Although the drug is associated with good outcomes in cancer, patients can develop inflammatory adverse events caused by immune activation, which are referred to as immune-related adverse events (irAE). Uveitis is a common irAE in ophthalmology [2], and severe cases of Vogt–Koyanagi–Harada disease (VKH)-like uveitis have been reported. Based on the uveitis guidelines in Japan, high-dose steroid therapy is used for VKH [3]. In most cases of ICI-induced VKH disease-like uveitis, both domestically and internationally, the same treatment strategy is used to date. However, high-dose steroid therapy can be challenging in some patients, such as elderly individuals and those with diabetes. Herein, we report a patient with nivolumab-induced VKH disease-like uveitis who had good outcomes after receiving treatment without intravenous methylprednisolone therapy.

## 2. Case Presentation

**Figure 1:**
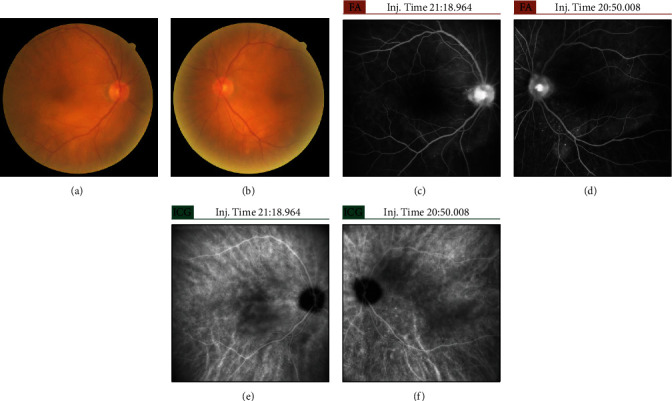
Color fundus photography, fluorescein angiography, and indocyanine green fluorescence angiography before treatment. The left column shows images of the right eye, and the right column shows images of the left eye. (a, b) Color fundus photography revealed extensive SRD and wavy RPE in both eyes. (c, d) Fluorescein angiography showed leakage at some pinpoint-sized areas, pooling on the posterior pole, and hyperfluorescent optic disks in both eyes. (e, f) Indocyanine green fluorescence angiography showed some hypofluorescent dark spots during the late phase.

**Figure 2:**
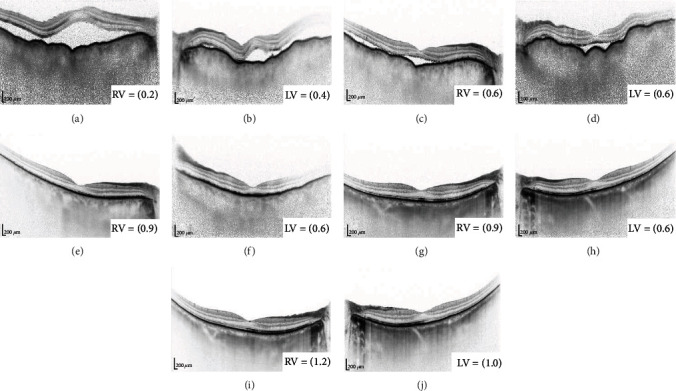
Images of treatment progress on optical coherence tomography. The left column shows images of the right eye, and the right column shows images of the left eye. The patient's decimal BCVA on the same day was obtained. (a, b) Before treatment, both eyes had SRD, wavy RPE, and choroidal thickening. (c, d) After 1 week, the volume of subretinal fluid decreased. Only the right eye received subtenon injection of triamcinolone acetonide. Both eyes had similar improvements in abnormal findings. (e, f) After 1 month, SRD and wavy RPE disappeared. At this point, the maximum visual acuity was achieved before cataract surgery. (g, h) After 6 months and a half, the patient was in remission. Choroidal thinning had become more prominent. (i, j) After approximately 1 year, the patient remained in remission.

A 72-year-old man was referred to our department due to bilateral blurry vision in June 2021. His decimal best-corrected visual acuities (BCVAs) were 0.2 in the right eye and 0.4 in the left eye. He had no previous medical history of infection, headache, tinnitus, hearing impairments, and vitiligo. Slit lamp examination revealed the presence of minimal bilateral anterior chamber cells and keratic precipitates. Fundus examination and optical coherence tomography showed serous retinal detachment (SRD), wavy retinal pigment epithelium (RPE), and choroidal thickening in both eyes (Figures 1(a), 1(b), 2(a), and 2(b)). Fluorescein angiography revealed leakage at some pinpoint-sized areas, pooling on the posterior pole, and hyperfluorescent optic disks in both eyes (Figures 1(c) and 1(d)). Indocyanine green fluorescence angiography showed some hypofluorescent dark spots during the late phase (Figures 1(e) and 1(f)). Cerebrospinal fluid examination was performed at the department of neurology, and results showed that the number of cells, predominantly mononuclear cells, increased to 142/*μ*L. Audiometry was conducted at the department of otorhinolaryngology. Based on the findings, presbyacusis was suspected. Human leukocyte antigen (HLA) typing revealed A2, A24, B35, B54, and DR4.

The patient was diagnosed with gastric cancer in 2019, and treatment with nivolumab was initiated in March 2021. However, in June 2021, 2 weeks before our initial examination, he developed fulminant type 1 diabetes mellitus and diabetic ketoacidosis after eight cycles of nivolumab therapy. We assumed that VKH disease-like uveitis was caused by nivolumab. Thus, nivolumab therapy was discontinued.

**Figure 3:**
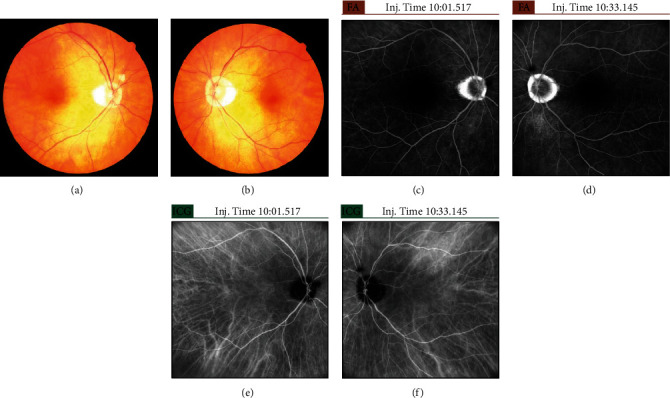
Color fundus photography, fluorescein angiography, and indocyanine green fluorescence angiography after treatment. The left column shows images of the right eye, and the right column shows images of the left eye. (a, b) Color fundus photography showed a sunset glow appearance. SRD and wavy RPE were not observed. (c–f) Abnormal findings on fluorescein angiography or indocyanine green fluorescence angiography had disappeared.

Next, treatment with topical corticosteroid (betamethasone sodium phosphate 0.1%) six times a day and tropicamide phenylephrine hydrochloride three times a day was started on both eyes. On the following day, the right eye, which had severe vision loss, received subtenon injection of triamcinolone acetonide (STTA). After 1 week, the patient's BCVA improved up to 0.6 in both eyes. However, there were no changes in SRD. Further, the volume of subretinal fluid decreased (Figures 2(c) and 2(d)). Both eyes had similar improvements in visual acuity and abnormal findings. After 1 month, SRD and wavy RPE disappeared (Figures 2(e) and 2(f)), and the BCVAs improved up to 0.9 in the right eye and 0.6 in the left eye. The ophthalmologic progress was good. However, the patient had hearing loss 2 weeks after the initial examination. The surgeon initiated treatment with oral prednisolone (40 mg) with tapering in August 2021. Hearing loss immediately improved, and oral steroid treatment was completed in October 2021. After oral steroid therapy, there was no improvement in BCVA. In both eyes, choroidal thinning (Figures 2(g) and 2(h)) and cataract (particularly in the left eye) gradually progressed. In February 2022, uveal inflammation was not observed, and bilateral cataract surgery (phacoemulsification and intraocular lens implantation) with STTA was performed. The BCVAs on the day after each surgery were 1.0 in the right eye and 1.2 in the left eye. After approximately 1 year after disease onset, abnormal findings such as leakage and pooling on fluorescein angiography and hypofluorescent dark spots on indocyanine green fluorescence angiography had disappeared (Figures 3(c)–3(f)). Fundus examination revealed a sunset glow appearance (Figures 3(a) and 3(b)). SRD and wavy RPE did not relapse 1 year after disease onset (Figures 2(i) and 2(j)).

## 3. Discussion

VKH is severe bilateral granulomatous posterior or panuveitis associated with SRD, disk edema, and vitritis [4]. It is an autoimmune inflammatory disease caused by cytotoxic T lymphocytes targeting melanocytes [5, 6]. Genetic phenotype and viral infection are involved in the pathogenesis of the disease [7], and several cases of VKH disease-like uveitis after the initiation of ICI, which is often used in cancer treatment in recent years, have been reported. There are 15 cases of nivolumab-induced VKH disease-like uveitis, including ours [8–20] (Tables 1 and 2).

Ophthalmic irAE are diverse, and the most common of which is uveitis (15.1%) [2]. Among all types of uveitis, the most frequently observed is anterior uveitis (37.7%) [21]. However, VKH disease-like findings are also observed in some cases. The median time from the start of ICI to the appearance of uveitis is 63 days, with 83.6% of cases appearing within 6 months [21]. In our case, fulminant type 1 diabetes mellitus, which is an irAE, appeared at the 106th day (15 weeks, 3.5 months), and ophthalmic symptoms at the 119th day (17 weeks, 4 months). Based on other reports of nivolumab-induced VKH disease-like uveitis, the onset was within 6 months (from 2 weeks to 4 months) in all but one case [12].

In patients with VKH, mononuclear cell-dominant cerebrospinal fluid pleocytosis, which is indicative of aseptic meningitis, is often observed, with a frequency of 82.7% [22]. A previous report did not show pleocytosis, thereby indicating the risk of a different etiology from the usual VKH [16]. However, in our case, the patient presented with prominent pleocytosis and VKH.

HLA-DR4, particularly DRB1^∗^0405, is associated with VKH [4, 23]. Of nine patients who underwent examination of HLA types, 7 (77.8%), including ours, had positive results. The frequency of the HLA-DR4 phenotype in Japanese patients with VKH is 81.6% [24]. In Southern California, the frequency is 56% [25]. Thus, a high proportion of patients with nivolumab-induced VKH disease-like uveitis and VKH present with HLA-DR4.

If irAE is suspected, ICI therapy should be discontinued. Based on the American Society of Clinical Oncology clinical practice guidelines, ICI should be permanently discontinued if posterior uveitis or panuveitis develops [26]. In clinical practice, ICI therapy is continued or resumed based on the extent of the underlying disease and irAE in some cases. Nivolumab treatment was discontinued in almost all cases since 2018, after the abovementioned guideline was published. In one case, the patient presented with choroidal thickening and anterior segment inflammation after restarting nivolumab treatment [9]. Hence, caution must be observed when continuing or resuming nivolumab.

Initially, acute VKH must be treated aggressively with corticosteroids, and local treatment alone is not recommended for this disease [4]. As shown in Table 2, the initial treatment for nivolumab-induced VKH disease-like uveitis is not certain, and whether systemic therapy, particularly intravenous methylprednisolone, is necessary is a matter of debate. Ours and previous cases showed that intravenous steroids (150–1,000 mg/day) were used in 8 of 15 patients. Moreover, 13 patients received systemic steroids, including oral steroids. Steroid therapy can cause complications, such as infection and diabetes, and its application in elderly individuals may be challenging. Nevertheless, there are several cases, including ours, in which systemic complications could not be controlled by local therapy alone. Systemic steroids may be important as in VKH. In addition, the extent of VKH disease-like findings in these reports varies. Ultimately, decision-making must be made on a case-to-case basis.

STTA was administered to only one eye, and it was observed that there was no difference in final visual acuity after 1 week of administration or in final visual acuity. To the best of our knowledge, this is the first case in which STTA was administered to one eye only. Prior to the initiation of oral steroids, SRD and wavy RPE disappeared, and the patient had the maximum BCVA before cataract surgery. Hence, remission was achieved with local therapy (eye drops and STTA) alone. There have been no recurrences since then. However, oral steroids administered for hearing loss could have prevented recurrence.

Nevertheless, whether nivolumab-induced VKH disease-like uveitis is different from diseases such as nivolumab-induced VKH and VKH is inconclusive. If the latter is considered, intravenous methylprednisolone therapy should be initiated, as in VKH. However, in a previous case, SRD resolved immediately after treatment with eye drops and STTA alone. Further, the BCVA improved, which is not commonly observed in normal VKH. Nevertheless, our patient required oral steroids due to extraocular complications. Hence, the development of a treatment regimen for nivolumab-induced VKH disease-like uveitis, with consideration of the systemic management and prevention of recurrence, remains a challenge.

## Figures and Tables

**Table 1:** Previous cases of nivolumab-induced Vogt–Koyanagi–Harada disease-like uveitis.

Authors	Year of publication	Age	Sex	Primary disease	ICI	Timing	Pleocytosis	HLA class II examination
Current case		72	M	Gastric cancer	Nivolumab	17 weeks	+ (142/*μ*L)	HLA-DR4
Hwang et al. [8]	2022	61	F	Ovarian cancer	Nivolumab	12–16 weeks	NR	NR
Minami et al. [9]	2021	73	M	Metastatic melanoma	Nivolumab Ipilimumab	25 days	NR	HLA-DR4
Ushio et al. [10]	2021	58	M	Non-small-cell lung cancer	Nivolumab	4 months	+ (16/*μ*L)	HLA-DR4
Godse et al. [11]	2021	57	F	Metastatic melanoma	Nivolumab Ipilimumab	3 months	NR	NR
Ng et al. [12]	2021	49	F	Metastatic renal cell carcinoma	Nivolumab Ipilimumab	2 years	NR	NR
Czichos et al. [13]	2021	70	M	Metastatic renal cell carcinoma	Nivolumab		NR	None
Mihailovic et al. [14]	2020	68	F	Metastatic melanoma	Nivolumab Ipilimumab		NR	NR
Gambichler et al. [15]	2020	63	F	Metastatic melanoma	Nivolumab		NR	NR
Kikuchi et al. [16]	2020	69	M	Hypopharyngeal cancer	Nivolumab	2 months	—	HLA-DRB1^∗^04:05 HLA-DRB1^∗^09:01
Obata et al. [17]	2019	63	F	Metastatic melanoma	Nivolumab	1 month and 10 days	NR	HLA-DR9
Fujimura et al. [18]	2018	73	M	Metastatic melanoma	Nivolumab Dabrafenib Trametinib		NR	HLA-DRB1^∗^04:05
Fujimura et al. [18]	2017	35	F	Metastatic melanoma	Nivolumab Dabrafenib Trametinib		NR	HLA-DRB1^∗^04:05
Arai et al. [19]	2017	55	M	Metastatic melanoma	Nivolumab	2 weeks	NR	HLA-DRB1^∗^04:10 HLA-DRB1^∗^04:06
Matsuo et al. [20]	2017	60	F	Metastatic melanoma	Nivolumab Vemurafenib	6 weeks	NR	NR

NR: not reported; F: Female; M: Male; ICI: immune checkpoint inhibitors; HLA: human leukocyte antigen.

**Table 2:** Treatments and outcomes in previous cases of nivolumab-induced Vogt–Koyanagi–Harada disease-like uveitis.

Authors	Nivolumab	Initial treatment (focal)			Initial treatment (systemic)	Outcome	Second treatment	Outcome
		STTA	Topical steroids	Mydriatics				
Current case	Discontinued	Unilateral	+	+	-	Improved, but with hearing loss	PSL 40 mg	Improved hearing loss
Hwang et al. [8]	Discontinued	NR	NR	NR	PSL 1,000 mg/day	Improved		
Minami et al. [9]	Discontinued	Bilateral	+	NR	mPSL 1,000 mg/day	Improved		
Ushio et al. [10]	Discontinued	NR	+	NR	-	Relapse	PSL 150 mg/day	Improved
Godse et al. [11]	Discontinued	NR	NR	NR	PSL 1 mg/kg/day	Improved		
Ng et al. [12]	Discontinued	-	-	-	-	Worsening	PSL 60 mg/day	Improved
Czichos et al. [13]	Discontinued	NR	+	+	PSL 200 mg/day	Improved		
Mihailovic et al. [14]	Continued	NR	+	+	PSL 2 mg/kg/day	Improved, but with macular edema	Intravitreal triamcinolone 2 mg	Improved
Gambichler et al. [15]	Discontinued	NR	NR	NR	mPSL 1,000 mg/day	Improved		
Kikuchi et al. [16]	Discontinued	Bilateral	NR	NR	-	No remission	mPSL 1,000 mg/day	Improved
Obata et al. [17]	Discontinued	NR	+	+	-	Improved		
Fujimura et al. [18]	Continued	NR	NR	NR	mPSL 500 mg/day	Improved		
Fujimura et al. [18]	Continued	NR	NR	NR	mPSL 500 mg/day	Improved		
Arai et al. [19]	Continued	NR	+	+	-	Improved		
Matsuo et al. [20]	Continued	NR	+	NR	PSL 30 mg	Improved		

NR: not reported; STTA: subtenon injection of triamcinolone acetonide; PSL: prednisolone; mPSL: methylprednisolone.

## Data Availability

All data used to support the conclusions of the study are available upon request.
